# Correlation between *Ureaplasma* Subgroup 2 and Genitourinary Tract Disease Outcomes Revealed by an Expanded Multilocus Sequence Typing (eMLST) Scheme

**DOI:** 10.1371/journal.pone.0104347

**Published:** 2014-08-05

**Authors:** Jun Zhang, Yingying Kong, Zhi Ruan, Jun Huang, Tiejun Song, Jingjuan Song, Yan Jiang, Yunsong Yu, Xinyou Xie

**Affiliations:** 1 Department of Clinical Laboratory, Sir Run Run Shaw Hospital, College of Medicine, Zhejiang University, Hangzhou, Zhejiang, China; 2 Biomedical Research Center, Sir Run Run Shaw Hospital, College of Medicine, Zhejiang University, Hangzhou, Zhejiang, China; University of Ulster, United Kingdom

## Abstract

The multilocus sequence typing (MLST) scheme of *Ureaplasma* based on four housekeeping genes (*ftsH*, *rpL22*, *valS*, and *thrS*) was described in our previous study; here we introduced an expanded MLST (eMLST) scheme with improved discriminatory power, which was developed by adding two putative virulence genes (*ureG* and *mba-np1*) to the original MLST scheme. To evaluate the discriminatory power of eMLST, a total of 14 reference strains of *Ureaplasma* serovars and 269 clinical strains (134 isolated from symptomatic patients and 135 obtained from asymptomatic persons) were investigated. Our study confirmed that all 14 serotype strains could successfully be differentiated into 14 eMLST STs (eSTs), while some of them could not even be differentiated by the MLST, and a total of 136 eSTs were identified among the clinical isolates we investigated. In addition, phylogenetic analysis indicated that two genetically significantly distant clusters (cluster I and II) were revealed and most clinical isolates were located in cluster I. These findings were in accordance with and further support for the concept of two well-known genetic lineages (*Ureaplasma parvum* and *Ureaplasma urealyticum*) in our previous study. Interestingly, although both clusters were associated with clinical manifestation, the sub-group 2 of cluster II had pronounced and adverse effect on patients and might be a potential risk factor for clinical outcomes. In conclusion, the eMLST scheme offers investigators a highly discriminative typing tool that is capable for precise epidemiological investigations and clinical relevance of *Ureaplasma*.

## Introduction

*Ureaplasma* is a member of the class *Mollicutes* and one of the smallest free-living organisms. It lacks a cell wall, displays limited biosynthetic abilities, requires cholesterol and hydrolyzes urea as a metabolic substrate to generate ATP [1]. To date, *Ureaplasma* is subtyped into 14 serovars that can be reclassified into two species. *Ureaplasma parvum* (UPA) includes 4 serovars (UPA1, UPA3, UPA6, and UPA14), while *Ureaplasma urealyticum* (UUR) comprises the remaining 10 serovars (UUR2, UUR4, UUR5, UUR7, UUR8, UUR9, UUR10, UUR11, UUR12, and UUR13) [2]. Genome sizes of UPA are between 0.75–0.78 Mbp and those of UUR are between 0.84–0.95 Mbp [1], [3].

*Ureaplasma* is regarded as a commensal organism in the urogenital tract of sexually active adults and the colonization rate of *Ureaplasma* has been found between 40 to 80% in female [4]. It is always implicated in many diseases including inflammation, non-gonococcal urethritis, chorioamnionitis, adverse pregnancy outcomes, infertility, bronchopulmonary dysplasia in neonates, etc. [5]–[10].

Why *Ureaplasma* are commensal organisms in some instances and arouse clinical manifestation in others? Whether there are any associations of particular species or serovars to clinical manifestations? Although many attempts are tried, the pathogenesis of *Ureaplasma* induced reverse outcome is still not yet clear.

For investigating the epidemiology of *Ureaplasma*, several molecular subtyping methods have been developed, including traditional PCR for species or serovars determination, restriction fragment length polymorphism (RFLP), pulsed field gel electrophoresis (PFGE) and real-time PCR [2], [11]–[13]. Recently, a multilocus sequence typing (MLST) scheme with four housekeeping genes has been established and verified by Zhang et al. [14] Compared to any other molecular subtyping method, MLST is more sensitive, specific, and reproducible. Moreover, MLST is a well-accepted way for illustrating the diversity and population structure of different bacterial species [15], [16]. Recent studies have displayed that virulence genes may provide genetic markers additionally [17]–[21]. MLST with antigen gene sequences has been developed to investigate the isolates involved in meningococcal disease [17].

During recent years, some studies reported that the putative pathogenic genes or proteins of *Ureaplasma* may be responsible for clinical outcomes [1], [3]. It contains multiple banded antigen (MBA) and its paralogous proteins, urease, phospholipase C, A1, and A2 (PLC, PLA1, PLA2), immunoglobulin-a (IgA) protease, nucleases, putative O-sialoglycoprotein peptidase, macrophage infection mutant protein (MimD), resisting hostile environment, etc. Compared to housekeeping genes, virulence genes diverse more rapidly and can be included for phylogeny studies of *Ureaplasma*. Thus, combination of housekeeping genes and virulence genes may be more suitable for illustrating the rationale of *Ureaplasma* being associated with clinical outcomes.

In our previous study, a total of 99 sequence types (STs) were identified from 14 reference strains and 269 clinical isolates according to the MLST data analysis. In addition, clonal complex (CC) 2 was found to be more frequently associated with symptomatic patients of diseases. Although it had the discriminating capacity to differentiate two biovars of *Ureaplasma*, the main drawback was it could not successfully differentiating all the 14 serovars from each other and the specific clinical relevance of these subgroups in CC2 has not yet been clearly established. Against this background, we now describe an updated and expanded six gene version of MLST scheme based on four housekeeping genes and two putative virulence genes (eMLST) that provides improved high resolution typing, in which the partial sequences from two putative virulence genes, namely an Urease complex component (*ureG*) and a MBA N-terminal paralog (*mba*-*np1*) were added. This six-gene based version (2814 bp) demonstrates greater levels of resolution compared to the original MLST scheme when analysed against a panel of 14 serovars of *Ureaplasma* and 269 clinical isolates.

## Materials and Methods

### Bacterial strains and clinical specimens

All the 283 strains have been analyzed in our previous study for developing a MLST scheme [14]. It contained 14 reference strains (UPA1, UPA3, UPA6, UPA14, UUR2, UUR4, UUR5, UUR7, UUR8, UUR9, UUR10, UUR11, UUR12, and UUR13) and 269 clinical strains (134 obtained from symptomatic patients and 135 isolated from asymptomatic persons). Mycoplasma IST2 (bioMérieux, Marcy l'Etoile, France) was used for the isolation of *Mycoplasma hominis* and *Ureaplasma*. All the 269 clinical strains of *Ureaplasma* were subcultured to A7 Mycoplasma agar plates (Kailin, Jiangmen, China) and grown in 10B urea broth (Liming, Nanjing, China) subsequently for purification of clinical specimens. All the stains were incubated at 37°C in an 5% CO_2_ atmosphere.

Our previous MLST scheme mentioned that 12 sequence types (STs) appeared in the 14 type strains and 87 novel STs emerged in 269 clinical isolates. As the predominant STs, ST1 and ST22 included 68 and 70 isolates, respectively. Besides that, two clonal lineages (CC1 and CC2) were revealed by eBURST software, and CC1 consisted the vast majority of clinical isolates. Moreover, we further confirmed the biotypes of clinical isolates, and found that isolates of CC1 were UPA and those of CC2 were UUR [14].

### DNA extraction

Zero point five milliliters (0.5 mL) of *Ureaplasma* broth culture of each *Ureaplasma* strain was harvested by centrifugation at 12,000× g for 10 min. 50 µL of lysis buffer (10 mM Tris-HCl, pH 8.0; 50 mM KCl; 2.5 mM MgCl_2_; and 0.5% Tween 20) and proteinase K (10 mg/mL) were used to resuspend the cell. The mixture was incubated at 55°C for 1 h and then heated at 95°C for 10 min. The sample was centrifuged at 10,000× g for 1 min to remove debris. The supernatant was utilized immediately or stored at −20°C for future use.

### Choice of loci for eMLST

The serovar 10 str. ATCC 33699 was used as reference and its gene sequences were retrieved from the genbank file (accession no. CP001184.1). The gene sequences were searched against the genome sequence of other *Ureaplasma* serovars by using BLAST tool. The accession number of these genome sequences was ABES00000000.1 (UPA1), ABFL00000000.2 (UUR2), CP000942.1 (UPA3), AAYO00000000.2 (UUR4), AAZR00000000.1 (UUR5), AAZQ00000000.1 (UPA6), AAYP00000000.1 (UUR7), AAYN00000000.2 (UUR8), AAYQ00000000.2 (UUR9), AAZS00000000.1 (UUR11), AAZT00000000.1 (UUR12), ABEV00000000.1 (UUR13), ABER00000000.1 (UPA14), respectively. The alignment threshold was set as e value lower than 1e-10. A total of 258 genes were found to be conserved among the 14 serovars. Of them, *ureG* (UU429) and *mba-np1* (UU485) showed great nucleotide diversity and were reported previously with virulence: *ureG* is one of urease complex components that metabolizes urea to generate energy production of ammonia and causes pathogenic effect [22], [23], and *mba-np1* is a paralogous gene of the multiple banded antigen (MBA) that might be involved in strategies for escaping the host immune system [24], [25]. Hence, *ureG* and *mba-np1* were chosen as the virulence loci for the eMLST scheme. The four previously described genes, i.e., *ftsH*, *rpL22*, *valS*, and *thrS*, were still involved in this study as the housekeeping loci.

### Amplification and DNA sequencing

Amplifications were performed according to Taq™ DNA Polymerases (Takara, Japan) protocol. Total volume of 50 µL contained 4 µL template DNA, 1× PCR Buffer (Mg^2+^ Free), 2.5 mM MgCl_2_, 2.5 mM dNTP Mixture, 0.25 µM of each PCR primer, and 0.5 U of Taq DNA polymerase. The PCR amplification was initiated with DNA denaturation at 94°C for 5 min, followed by 35 cycles of amplification at 94°C for 30 s, 50°C for 30 s, 72°C for 1 min, and with a final extension period at 72°C for 5 min, then cooling to 10°C.

The purification of PCR products was carried out by adding 2 volumes of 95% ethanol and 0.1 volumes of 2.5 M NaCl. The mixture was placed in −20°C for 1 h then centrifuged and the remainder pellet was washed with 70% ethanol. ABI 3730xl DNA analyzer was used for sequencing of the purified PCR products, according to the manufacturer's instructions. The primers for the amplification and sequencing of the six loci are displayed in Table 1.

**Table 1:** Primer sequences, size of fragment, and percent coverage of complete coding sequence for six loci in this study.

Gene	Annotation	Size (bp) of fragments Analyzed	Coverage of complete CDS (%)	PCR and sequence primers (5′-3′) (F/R)^a^	Reference
*ftsH*	Cell division protein FtsH	463	21.38	TAAAAAAGACGACTTAACTCAACC (F)AATAAAGAGTCGCTTTGTGCT (R)	14
*rpL22*	50S ribosomal protein L22	456	48.71	TCCAACAATGAAAAGAACACT (F)TTTTCCTTCATAGTAAGCATC (R)	14
*valS*	Valyl-tRNA synthetase	335	12.76	GTCTCAAGAATGATGAACTTTAGCC (F)GCAACAACTAGATTATATTTATCC (R)	14
*thrS*	Threonyl-tRNA synthetase	598	34.31	TGATACTGTTATTACGCCTATA (F)AGCGGTAAAATACCTTTAGTTTGTT (R)	14
*ureG*	Urease complex component	482	77.62	TTAATTATTGGTGTAGGTGGACCTG (F)TCAATTCAATCAGCAACAGAT (R)	This study
*mba-np1*	MBA N-terminal paralog	480	22.66	TAGCGGATTTATCGGTTGAACTATA (F)TTAGTTTCAGCACGCCAACCATC (R)	This study

a F indicates forward primer and R indicates reverse primer.

### Allele and sequence type assignment

The Molecular Evolutionary Genetic Analysis software (MEGA version 5.0) was used to perform multiple sequence alignments [26]. Different allelic types (ATs) (sequences with at least a one-nucleotide difference or one-spacer difference) were designated arbitrary numbers. Each allelic profile was determined by the corresponding combination of six alleles (*ftsH*, *rpL22*, *valS*, *thrS*, *ureG*, *and mba-np1*) and assigned an unique eMLST Sequence Type (eST). Novel alleles for each locus were assigned a new allele number and distinct allelic profiles assigned a new eST number.

### Analysis of allelic polymorphism

The software named Sequence Type Analysis and Recombinational Tests (START2, http://pubmlst.org/software/analysis/start2/) was used to analyze allelic profiles, number of polymorphic nucleotide sites, and G+C content [27]. A useful measurement for identifying adaptive protein evolution is the nonsynonymous (*dN*)/synonymous substitution (*dS*) rate (*ω* = *dN*/*dS*), where values of *ω* = 1, <1, and >1 indicate neutral selection, negative selection, and positive selection, respectively. The nucleotide diversity per site (θ), the average number of nucleotide differences per site (π), Tajima's *D* test were determined using DnaSpv5 software [28], which is based on the differences between the θ and π values. Discrimination index (D.I.) values were calculated on the basis of numbers of allelic types [j], numbers of strains belonging to each type [n_j_], and total numbers of strains analyzed [N]) with the following equation [29]. Higher D.I. value indicates higher discriminatory power.
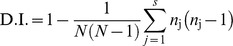



### Codon-based analyses of positive selection

Evidence for recombination breakpoints was assessed using the genetic algorithm detection (GARD) method and individual codons were analyzed for positive selection using the following methods: Single Likelihood Ancestor Counting (SLAC), the Fixed Effect Likelihood (FEL), the Random Effect Likelihood (REL), and the Mixed Effects Model of Evolution (MEME) of HyPhy software, which were implemented in the Datamonkey web server and applied REV and HKY85 models of nucleotide substitution [30]–[32]. To avoid a high false-positive rate, sites with *p* values <0.1 for SLAC, FEL and MEME models, and Bayes Factor >50 for REL model were accepted as candidates for selection.

### Phylogenetic analysis

A Neighbor-Joining tree of *Ureaplasma* isolates was constructed by MEGA 5.0 using the number of nucleotide differences in the concatenated sequences (total of 2814 bp) of six loci, with 1,000 bootstrap tests. To visualize the large data better, we selectively compressed subtrees with genetically similar isolates using the Compress/Expand function in MEGA 5.0.

### Statistical analysis

IBM SPSS Statistics 19.0 was used to analyze the association between sub-groups and diseases, based on the Chi-square test. The *p* values<0.05 were considered statistically significant.

## Results

### Levels of genetic diversity between eMLST loci

On the basis of four loci selected in the MLST for *Ureaplasma*, we added two putative virulence loci (*ureG* and *mba-np1*) to develop an eMLST scheme. The sizes of *ureG* and *mba-np1* partial sequences analyzed in this study were 482 bp and 480 bp, respectively. While the *ftsH* and *mba*-*np1* genes had the highest number of polymorphic sites due to greater quantity of alleles. The number of alleles per locus ranged from 10 (*rpL22* and *ureG*) to 39 (*ftsH*), while the number of polymorphic sites ranged from 14 (3.07%; *rpL22*) to 138 (29.81%; *ftsH*). The discriminatory index (D.I.) was the lowest for *valS* (33.6%) and the highest for *mba*-*np1* (93.4%). Moreover, in the 14 type strains of the *Ureaplasma* serovars, the *ureG* gene showed five alleles and 36 polymorphic sites and the *mba-np1* gene revealed ten alleles and 54 polymorphic sites, and in the 269 clinical isolates, the *ureG* gene displayed nine alleles and 40 polymorphic sites, and the *mba-np1* gene displayed 30 alleles and 95 polymorphic sites. Overall, the level of diversity was low as reflected in the θ and π values calculated for each gene, and all candidate loci had *dN/dS* <1 indicating stabilising selection. The Tamija's *D* value for all loci did not deviate significantly from zero (*p*>0.1) which was consistent with neutral (random) evolution and therefore suitable for eMLST analysis (Table 2).

**Table 2:** Loci characteristics and discriminatory power of eMLST schemes based on four housekeeping genes and two putative virulence genes.

Gene	No. alleles	No. polymorphic sites	% polymorphic sites	θ^a^	π^b^	*d_N_*/*d_S_*	G+C%	D.I.%^c^	Tajima's *D* test^d^
*ftsH*	39	138	29.81%	0.07219	0.07219	0.2898	26.62	60.63	−1.016292
*rpL22*	10	14	3.07%	0.01085	0.01131	0.5367	29.17	62.79	0.191345
*valS*	21	51	15.22%	0.04591	0.06578	0.1919	25.53	33.66	1.278539
*thrS*	17	74	12.37%	0.03809	0.04809	0.0595	29.67	38.95	0.309837
*ureG*	10	39	8.09%	0.0308	0.03725	0.0126	33.57	59.34	−0.274874
*mba-np1*	35	100	20.83%	0.04804	0.04041	0.2131	22.88	93.40	−0.33261

a Nucleotide diversity per site.

b Average number of nucleotide differences per site.

c D.I.: discriminatory index.

d *p*>0.1 for all Tajima's *D* test results.

### eMLST analysis of *Ureaplasma* and predominant eSTs

A total of 283 isolates could be classified into 146 eSTs by using the eMLST scheme. Among the 14 *Ureaplasma* serovars, each serovar type represented one eST; while in the 269 clinical isolates, 136 eSTs were revealed. Only four eSTs (eST1, eST2, eST4, and eST9) appeared in both reference and clinical strains. Moreover, 132 novel eSTs emerged in the clinical isolates.

Among the 269 clinical isolates analyzed, the distribution of 136 eSTs was found to be uneven. eST41 and eST16, as the predominant eSTs, included 51 and 41 isolates, respectively. Interestingly, all of the eST41 isolates belonged to ST22 and eST16 isolates belonged to ST1, which were identified by MLST scheme.

### Selective pressure test on two putative virulence loci

In order to demonstrate these two putative virulence loci: *ureG* and *mba-np1* were not under positive or diversifying selection, we conducted a number of further analyses. We firstly examined our data using the tests of selection described by Single Likelihood Ancestor Counting (SLAC) and Mixed Effects Model of Evolution (MEME) algorithms. Based on SLAC algorithm, no clear evidence of episodic diversifying selection could be found in either gene (*p*<0.1, threshold) by using both HKY85 and REV models of substitution under recommended cut-off values. However, for *mba-np1* gene, a total of six sites were identified as being under potential diversifying selection with both HKY85 and REV models under the MEME algorithm (*p*<0.1, threshold); this number reduced to zero at the threshold of *p*<0.01.

We also analyzed the *ureG* and *mba-np1*gene sequences using Fixed Effect Likelihood (FEL) and Random Effect Likelihood (REL) tests of selection. With both FEL and REL algorithms, there were also no clear evidence of sites under episodic diversifying selection which could be identified for either gene using both HKY85 and REV models of substitution (*p*<0.1, threshold). These analyses provided limited evidence for any positive selection to the two putative virulence genes. Although MEME algorithm highlighted six *mba-np1* gene sites that may be under episodic diversifying selection, the results were still inconsistent with many other tests of selection. Besides, we also created the generate phylogenetic trees, which based on the two putative virulence gene and four housekeeping gene sequences, and indicated that they were congruent with those based on housekeeping genes (Figure S1). On this basis, it is reasonable to believe that these genes were not under diversifying selection and have co-evolved with the housekeeping genes.

### Phylogenetic analyses

**Figure 1:**
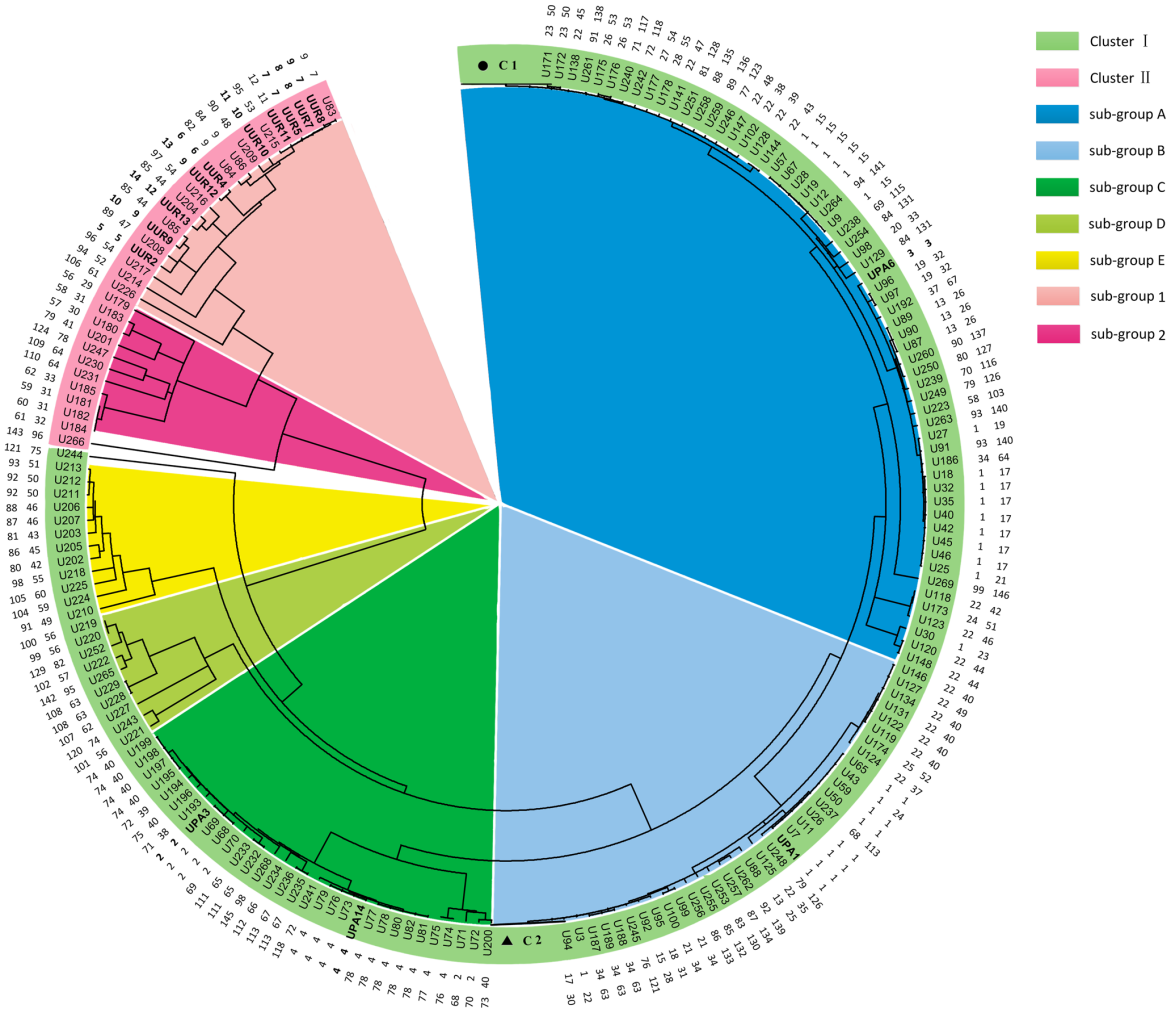
Neighbor-Joining tree was constructed by MEGA5.0 software, which based on concatenated nucleotide sequences of six loci. Two genetically significantly distant clusters were observed among the 283 strains. Additionally, five and two sub-groups were found in the cluster I and II, respectively. C1 included 54 isolates that represented eST41 (ST22), eST65 (ST35), eST66 (ST36) and eST144 (ST97). C2 embodied 44 isolates that stood for eST 16 (ST1), eST18 (ST1), eST20 (ST1) and eST29 (ST16). The isolates and the corresponding STs and eSTs are given at the tip of each branch. Two compressed subtree of C1 and C2 are highlighted in the *circle* and *triangle*, respectively. Bootstrap values str shown for 1,000 replicates.

As the *ureG* and *mba-np1*genes were under purifying selection and containing phylogenetically valuable information, they could be utilized in combination with the housekeeping genes for investigating the phylogenetic relationships within this species. To investigate the genetic relationship of isolates of *Ureaplasma*, a Neighbor-Joining tree, with 1,000 bootstrap replications, was constructed on the basis of the concatenated sequences of six gene fragments (Fig. 1). All the 283 isolates could be divided into two genetically significantly distant clusters. To visualize the large data set better, we selectively compressed subtrees with genetically similar isolates.

Among the 14 type strains of the *Ureaplasma* serovars, UPA (UPA1, UPA3, UPA6 and UPA14) existed in cluster I and UUR (UUR2, UUR4, UUR5, UUR7, UUR8, UUR9, UUR10, UUR11, UUR12 and UUR13) were present in cluster II. These results were highly congruent with previously identified genetic lineages. Among the 269 clinical isolates, cluster I constituted the overwhelming majority of 245 isolates and cluster II included only 24 isolates.

In the major cluster I, five sub-groups were observed and the member of each sub-group was high genetic similarity. Sub-group A contained 114 isolates (48 eSTs), sub-group B included 78 isolates (26 eSTs), sub-group C comprised 30 isolates (17 eSTs), sub-group D incorporated 10 isolates (10 eSTs), and sub-group E contained 12 isolates (11 eSTs). In the cluster II, sub-group 1 and 2 comprised 12 (11 eSTs) and 11 (11 eSTs) isolates, respectively.

### Comparison between MLST and eMLST

**Figure 2:**
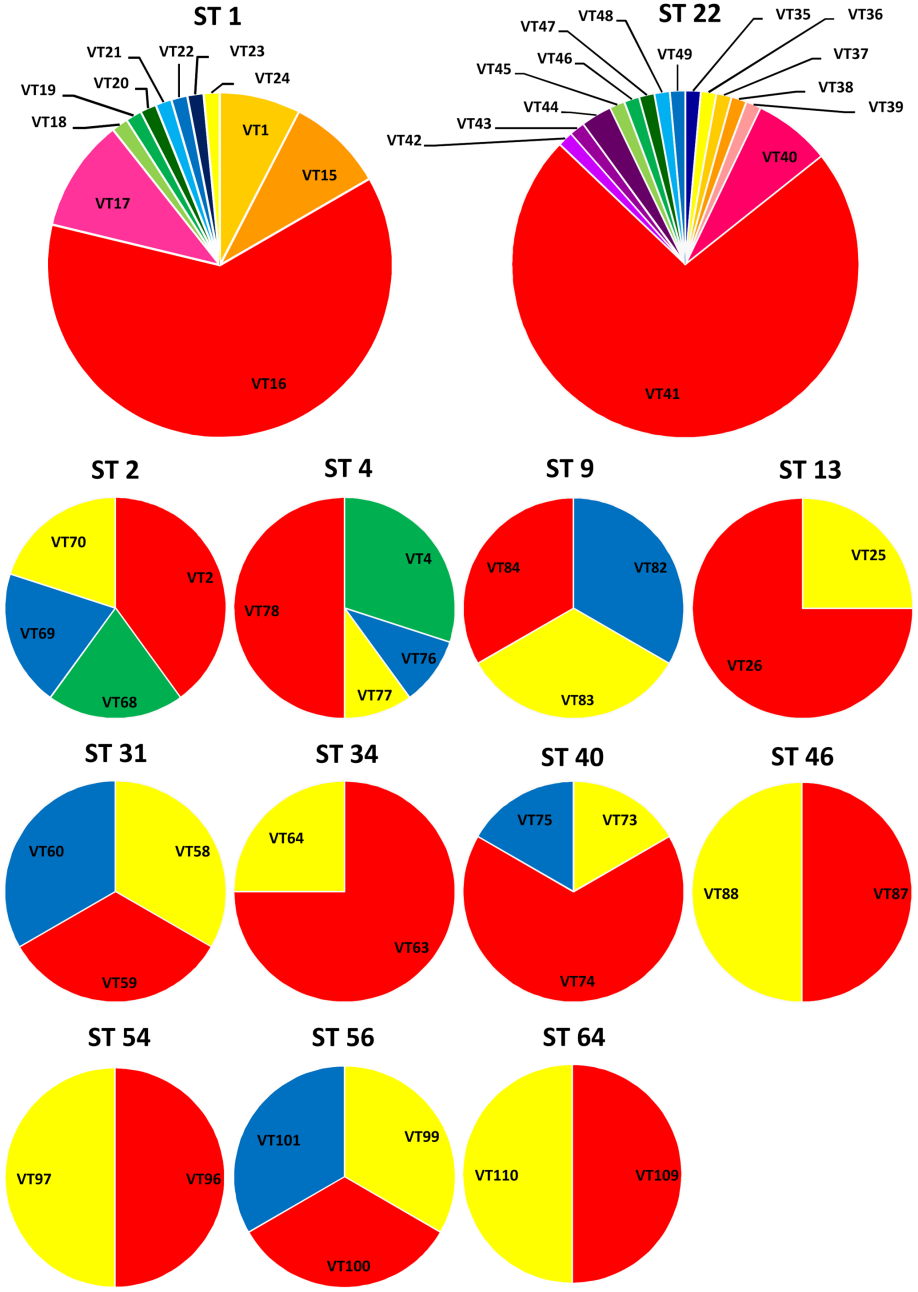
Comparison between STs and eSTs. 13 STs could be subdivided into two or more eSTs.

In our previous study, the 283 isolates were classified into 99 STs by using the MLST scheme [14], while a total of 146 eSTs were revealed in this study. According to the eMLST scheme, all 14 *Ureaplasma* serovars could successfully be differentiated into each other and were assigned to an unique eST, while some serovars (UUR5 and UUR8, UUR9 and UUR12) were not able to be differentiated using the MLST scheme. To better understand the discriminatory power of eMLST and MLST, we compared the eSTs and STs received form the corresponding scheme (Fig. 2), and 13 STs could be sub-divided into two or more eSTs. It was noteworthy that a total of 11 and 15 eSTs were revealed in the ST1 and ST22, which were the predominant STs. In addition, we also calculated the discriminatory index (95% confidence intervals) between two methods in terms of the 283 isolates analysed. Our data also indicated that results from discriminatory index value of MLST and eMLST methods were 86.9% (95% CI 83.9–89.9) and 93.9% (95% CI 92.0–95.9), respectively (*p*<0.001). While the eMLST scheme appears to demonstrate higher levels of resolution overall when compared to our previous MLST scheme.

### Association with symptomatic and asymptomatic infection

Five sub-groups and one singleton in cluster I and two sub-groups and one singleton in cluster II were selected for studying the association with symptoms (Table 3). We calculated the number and proportion of symptomatic infection and asymptomatic infection in all sub-groups and singletons. In the 134 clinical specimens obtained from symptomatic patients, there were 55 (41.04%), 38 (28.36%), 12 (8.96%), 4 (2.99%), and 7(5.22%) samples clustered in sub-group A, B, C, D, and E of cluster I; while 7 (5.22%) and 10 (7.46%) isolates cases belonged to sub-group 1 and 2, respectively. Obviously, sub-group A and B comprised of the bulk of strains analyzed. Compared to the asymptomatic infection, the rate of sub-group 2 was relatively higher in symptomatic infection (*p* = 0.005).

**Table 3:** Sub-groups of *Ureaplasma* in persons according to symptoms.

	Cluster I						Cluster II			Total
	Sub-group A	Sub-group B	Sub-group C	Sub-group D	Sub-group E	Singleton	Sub-group 1	Sub-group 2	Singleton	
Symptomatic infection	55 (41.04%)	38 (28.36%)	12 (8.96%)	4 (2.99%)	7 (5.22%)	0 (0)	7 (5.22%)	10 (7.46%)	1 (0.75%)	134
Asymptomatic infection	59 (43.70%)	40 (29.63%)	18 (13.33%)	6 (4.44%)	5 (3.70%)	1 (0.74%)	5 (3.70%)	1 (0.74%)	0 (0)	135
Sub-total	114	78	30	10	12	1	12	11	1	269
*p* value	0.659	0.818	0.254	0.527	0.546	1.000	0.546	**0.005** *	0.498	

*p<0.05 were displayed in **bold**.

## Discussion

As amplification and sequencing technology become increasing automated and available, MLST and eMLST will become more convenient and prompt for studying the epidemiology of *Ureaplasma*. Moreover, all the information can be submitted to internet-based databases for effortless comparison, thus a global epidemiological records will be generated. MLST scheme represents basic clonal assignments based on the variation in several housekeeping genes [15], [16], [33], whereas virulence genes can be adopted to “zoom in” on clones and differentiate very closed strains. Obviously, eMLST could provide a higher level of discrimination than MLST, based on the combination of housekeeping genes and virulence genes of this species, and therefore may be more appropriate for studying the epidemiology. For example, an eMLST scheme designed for *Propionibacterium acnes* is capable to differentiate pathogenic from non-pathogenic (commensal) strains and provides improved high resolution typing (91 eSTs from 285 isolates) to have important therapeutic and diagnostic implications [34].

In our previous study, we developed a MLST scheme that comprised four housekeeping genes for *Ureaplasma* [14]. Although it had highly discriminating capacity to differentiate the two biovars of *Ureaplasma*, it was inadequate to separate the 14 serovars and associate STs to clinical outcomes. To improve the resolution of this scheme, we increased the number of loci analysed. Due to the genome size of *Ureaplasma* (0.75∼0.95 Mbp; approximately 600 genes in the genome), the number of candidate loci for an expanded MLST scheme, especially house keeping loci, was very limited. As a result, we introduced two putative virulence genes (*ureG* and *mba-np1*) to the scheme as such genes are being increasing utilised in MLST schemes as they may be under positive selection, which can result in enhanced diversity and discriminatory power, and can also provide information on the evolution of virulence. In our study, the highly polymorphic sites and the low *dN/dS* ratio of *ureG* and *MBA-NP1* gene indicated that they were suitable for genetic analysis. Moreover, we also confirmed that these genes were not under diversifying selection and have co-evolved with housekeeping genes, and on this basis, we would consider them as a part of the core genome of *Ureaplasma*.

Herein, 146 eSTs were revealed in the 283 isolates investigated. A Neighbor-Joining tree was constructed with the purpose of understanding the genetic relationship better, and two genetically significantly distant clusters (cluster I and cluster II) were shown with very high internal bootstrap values in the 283 isolates. As expected, this assortment showed exact congruence with the two well-known genetic lineages (UPA and UUR) analyzed in our previous study [14]. Most *Ureaplasma* strains existed in the major cluster I and only a small portion was present in the cluster II. Among the cluster I and II, five and two sub-groups were found, respectively, and the members always owed high genetic similarity. It was noteworthy that UU244 (eST121) and UU266 (eST143) were found as singletons and the same results were received through the MLST scheme by presenting as ST75 and ST96, which might be due to the relatively higher variation in the selected loci.

In our present study, the higher discriminating capacity of eMLST scheme was revealed as the isolates with the same STs could be further divided into several eSTs. Two of the most striking cases were ST1 (68 isolates) and ST22 (70 isolates) that represented as 11 eSTs and 15 eSTs, respectively. Another significant and prominent instance was that the present eMLST scheme provided a clear discrimination of the 14 serotype strains, in which the 14 reference strains of *Ureaplasma* serovars were able to be separated into 14 eSTs and presented in the cluster I and cluster II accurately by simultaneously targeting two virulence and four housekeeping genes.

Up to now, there is a great controversy regarding the virulence of *Urealplasma* and the associations between species or serovars and diseases. With regard to relationship between species and bronchopulmonary dysplasia (BPD), different consequences received by researchers [5]–[7], [10]. In recent study, no significant difference was found in the incidence of *Ureaplasma* species regarding symptoms [35]. Additionally, our previous study found persons colonized with CC2 were prone to associate symptom while it cannot connect STs to clinical outcomes. In present study, our original objective was to correlate symptoms with the sub-groups or eSTs of isolates in consideration of the pathogenic genes might be in charge of the clinical manifestations. Both clusters were associated with clinical outcomes, and on this basis, symptom seemed to have no association with specific clusters. Compared to any other sub-groups, sub-group 2, a sub-group of cluster II, were more likely to colonize in symptomatic patients than asymptomatic persons. Colonization by sub-group 2 seemed to be a potential risk factor for clinical manifestations and needed for further investigation. As the strain numbers of most eSTs were very limited, it was difficult to draw any decisive conclusions on any possible correlations between eSTs and pathologies of *Ureaplasma*.

In conclusion, the expanded six gene MLST scheme greatly improved the discriminatory power of molecular epidemiology against a large panel of *Ureaplasma* isolates by adding two putative pathogenicity genes. Particularly, it enabled a more accurate differentiation scheme of all 14 reference serotypes and improved separation of 269 clinical isolates. Our data suggested that sub-group 2 were more likely to associate with clinical manifestations and further investigation was required to confirm it's clinically meaningful.

## Supporting Information

Figure S1 **Neighbor-Joining phylogenetic trees for four housekeeping genes** ***ftsH*** **(A),** ***rpL22*** **(B),** ***valS*** **(C) and** ***thrS*** **(D); two virulence genes** ***ureG*** **(E), and** ***mba*** **-** ***np1*** **(F).** All trees were essentially concordant with that previously obtained using four housekeeping loci, with the major divisions (Cluster I and II) forming the similar clades.
(TIF)
