# Supplementary figures and images for: Correlation between Ureaplasma Subgroup 2 and Genitourinary Tract Disease Outcomes Revealed by an Expanded Multilocus Sequence Typing (eMLST) Scheme
Source: PLoS One. 2014 Aug 5;9(8):e104347. doi: 10.1371/journal.pone.0104347 (PMC4122457; doi:10.1371/journal.pone.0104347)

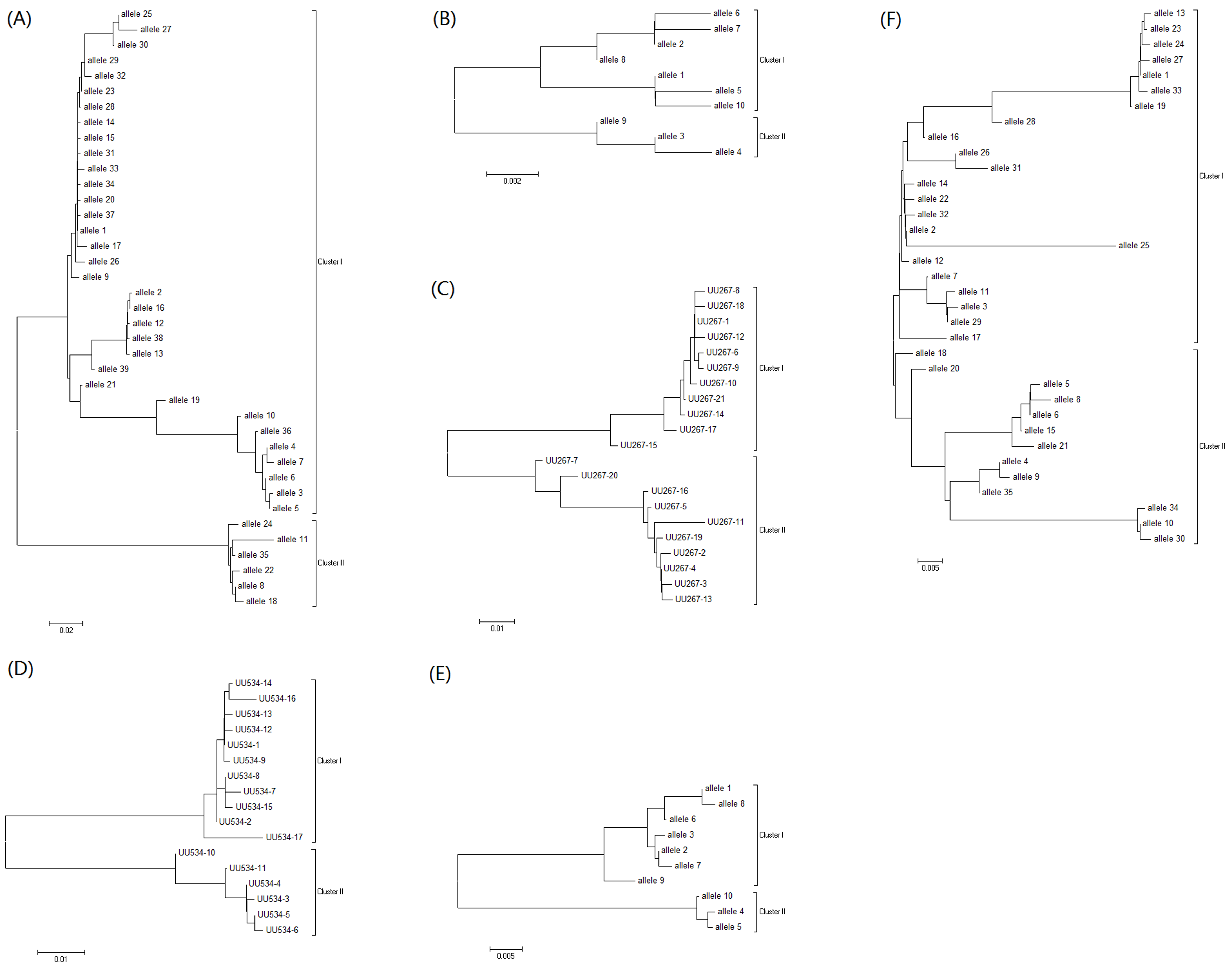

Supplement: Figure S1 — Neighbor-Joining phylogenetic trees for four housekeeping genes ftsH (A), rpL22 (B), valS (C) and thrS (D); two virulence genes ureG (E), and mba - np1 (F). All trees were essentially concordant with that previously obtained using four housekeeping loci, with the major divisions (Cluster I and II) forming the similar clades. (TIF) [file pone.0104347.s001.tif]
